# Myostatin Deficiency Protects C2C12 Cells from Oxidative Stress by Inhibiting Intrinsic Activation of Apoptosis

**DOI:** 10.3390/cells10071680

**Published:** 2021-07-03

**Authors:** Marius Drysch, Sonja Verena Schmidt, Mustafa Becerikli, Felix Reinkemeier, Stephanie Dittfeld, Johannes Maximilian Wagner, Mehran Dadras, Alexander Sogorski, Maxi von Glinski, Marcus Lehnhardt, Björn Behr, Christoph Wallner

**Affiliations:** Department of Plastic Surgery, BG University Hospital Bergmannsheil, Ruhr University Bochum, Bürkle-de-la-Camp Platz 1, 44789 Bochum, Germany; marius.drysch1@gmx.net (M.D.); sonja.schmidt@rub.de (S.V.S.); mustafa.becerikli@rub.de (M.B.); Felix.Reinkemeier@rub.de (F.R.); Stephanie.Dittfeld@bergmannsheil.de (S.D.); max.jay.wagner@googlemail.com (J.M.W.); Mehran.Dadras@bergmannsheil.de (M.D.); Alexander.Sogorski@bergmannsheil.de (A.S.); maxivonglinski@gmail.com (M.v.G.); marcus.lehnhardt@bergmannsheil.de (M.L.); christoph.wallner@bergmannsheil.de (C.W.)

**Keywords:** ischemia, reperfusion, hypoxia, reoxygenation, myostatin, GDF8, skeletal, muscle

## Abstract

Ischemia reperfusion (IR) injury remains an important topic in clinical medicine. While a multitude of prophylactic and therapeutic strategies have been proposed, recent studies have illuminated protective effects of myostatin inhibition. This study aims to elaborate on the intracellular pathways involved in myostatin signaling and to explore key proteins that convey protective effects in IR injury. We used CRISPR/Cas9 gene editing to introduce a myostatin (*Mstn*) deletion into a C2C12 cell line. In subsequent experiments, we evaluated overall cell death, activation of apoptotic pathways, ROS generation, lipid peroxidation, intracellular signaling via mitogen-activated protein kinases (MAPKs), cell migration, and cell proliferation under hypoxic conditions followed by reoxygenation to simulate an IR situation in vitro (hypoxia reoxygenation). It was found that mitogen-activated protein kinase kinase 3/6, also known as MAPK/ERK Kinase 3/6 (MEK3/6), and subsequent p38 MAPK activation were blunted in C2C12-*Mstn*^−/−^ cells in response to hypoxia reoxygenation (HR). Similarly, c-Jun N-terminal kinase (JNK) activation was negated. We also found the intrinsic activation of apoptosis to be more important in comparison with the extrinsic activation. Additionally, intercepting myostatin signaling mitigated apoptosis activation. Ultimately, this research validated protective effects of myostatin inhibition in HR and identified potential mediators worth further investigation. Intercepting myostatin signaling did not inhibit ROS generation overall but mitigated cellular injury. In particular, intrinsic activation of apoptosis origination from mitochondria was alleviated. This was presumably mediated by decreased activation of p38 caused by the diminished kinase activity increase of MEK3/6. Overall, this work provides important insights into HR signaling in C2C12-*Mstn*^−/−^ cells and could serve as basis for further research.

## 1. Introduction

Ischemia reperfusion (IR) injury remains a challenging topic in clinical medicine and has led to the proposal of several prophylactic and therapeutic strategies. While some of these include unspecific approaches that aim to stimulate a multitude of pathways (e.g., remote ischemic preconditioning [1]), several targeted therapies have been presented [2,3]. For their development, a thorough comprehension of involved molecular pathways is essential. In brief, circuits involved in the pathogenesis of IR injury are mainly initiated by adenosine triphosphate (ATP) depletion and reactive oxygen species (ROS) production [4]. Eventually, these cascades culminate in different forms of (programmed) cell death [4]. Besides classically known apoptosis and necrosis, the contribution of transitional (e.g., necroptosis) and additional (e.g., pyroptosis) forms has been described [4,5]. Crucial involvement has been attributed to the role of mitochondria. Depletion of ATP, elevated Ca^2+^ levels, and ROS production lead to an opening of the mitochondrial permeability transition pore (mPTP), which is a crucial determinant of cell death [6]. Interestingly, mitochondria are also the origin of the intrinsic apoptotic pathway [7]. In brief, the intrinsic apoptotic pathway is activated by release of cytochrome C from mitochondria, which is caused by an imbalance in proaptotic proteins (e.g., BAX, BAK) and antiapoptotic proteins (e.g., Bcl-2). Cytochrome C then binds Apaf-1, which leads to activation of caspase 9 and, subsequently, caspase 3. The extrinsic pathway of apoptosis is activated upon ligand binding to death receptors, which in turn leads to activation of caspase 8. Caspase 8 then activates further members of the caspase family and, eventually, caspase 3 [8,9,10]. As for intracellular signaling in terms of IR states, mitogen-activated protein kinases (MAPKs) play an important role. MAPKs mainly encompass three sub-categories, namely extracellular signal-regulated kinases (ERKs), c-Jun N-terminal kinases (JNKs), and p38 mitogen-activated protein kinases (p38s). While there is some overlap, ERKs in general are activated by growth factors and in terms of metabolic function. JNKs and p38s are responsible for transducing cellular stress signals from IR injury [11,12]. The main regulators of p38 activity are MEK3 and MEK6, which are kinases dedicated to the phosphorylation of p38 [13]. In addition, p38 is a downstream target of myostatin [14]. Myostatin was discovered in 1997 and its main function is the regulation of skeletal muscle mass [15]. Recent studies have shown that its role in cell signaling is not limited to muscle regulation. For instance, Verzola et al. [16] demonstrated that myostatin activation exhibits effects on vascular smooth muscle and inflammatory cells. An influence on bone regeneration has been demonstrated as well [17]. Moreover, interdependent regulation between myostatin and tumor necrosis factor alpha (TNF-α) as well as ROS activation have been illustrated [18]. Indeed, studies have investigated the protective capacity of intercepting myostatin signaling, and have shown that inhibiting myostatin reduced IR injury in murine cardiac as well as skeletal muscle cells [2,19]. However, the interplay between myostatin signaling, MAPK activity, ROS activation, and cell death has not been thoroughly evaluated, especially not in skeletal muscle. Therefore, this study aims to illuminate the role of myostatin signaling in skeletal muscle cells in response to HR/IR injury and to place it into the pathophysiological cascades of ROS generation and stress signaling via p38 and JNK that lead to cell death. For this reason, we used C2C12 cells and a CRISPR/Cas9 gene-edited C2C12 cell line in an in vitro HR/IR model.

## 2. Materials and Methods

### 2.1. Myoblast Culturing

Cell culture was carried out at a sterile cleanroom workbench. The myoblast mouse cell line C2C12 (ATCC cell culture bank) was acquired and incubated in a gassing incubator at 37 °C, 5% CO_2_, and a relative humidity of 97%. Cell growth and morphology were monitored with a phase contrast microscope (Axiovert 25, Zeiss, Jena, Germany). Cells were cultured in proliferation medium (DMEM, 10% FCS, 2% Na pyruvate, 1% NEAA, 1% Pen/Strep), which was changed every second day. Subculturing at 70% confluence was achieved by enzymatic digestion (0.05% trypsin/0.02% EDTA). Cell count and cell vitality were certified using a CASY cell counter (Roche, Basel, Switzerland). For experiments, C2C12 cells in plates with 24 or 6 wells (Sarstedt, Nümbrecht, Germany) with a cell density of 15,000 cells/cm^2^ were used.

### 2.2. Generation of a C2C12-Mstn^−/−^ Cell Line

After determining the DNA plasmid ratio, seeding of the C2C12 cells 24 h before co-transfection was performed according to the protocol of Santa Cruz. A total of 1.5 µg of GDF8-HDR plasmid (sc-421724-HDR, Santa Cruz, Dallas, TX, USA) in co-transfection with 1.5 µg of GDF8-CRISPR/CAS9 KO plasmid (sc-421724, Santa Cruz) was utilized for one well of a 6-well plate using 10 µL of Ultracruz transfection reagent (sc-395739, Santa Cruz). A change of medium was performed after 24 h, and co-transfection was confirmed by fluorescence microscopy. After adjusting the concentration, 10 µg/mL Puromycin (sc-108071, Santa Cruz) was added 24 h after co-transfection, and subsequent daily changes of the selection medium were performed. The selected C2C12 cells were trypsinized and dilutions with a concentration of 20 cells/100 μL were taken. Subsequently, 200 μL/well of this dilution was applied in the first row of a 96-well plate with subsequent serial dilutions [20]. Microscopic control was performed 24 h after incubation. The resulting cells were termed C2C12-*Mstn*^−/−^ cells.

### 2.3. IR/HR In Vitro Model

Cells were seeded in chamber slides with a density of 15,000 cells/cm^2^. After 24 h, cells were either incubated at normoxic conditions (21% O_2_, 5% CO_2_, 37 °C), resulting in the control group (C2C12-C cells or C2C12-*Mstn^−/−^*-C cells, respectively), or at hypoxic conditions (1% O_2_, 5% CO_2_, 94% N_2_, 37 °C) for 4 h followed by 15 min of reoxygenation (21% O_2_, 5% CO_2_, 37 °C) for the HR group (C2C12-HR cells or C2C12-*Mstn^−/−^*-HR cells, respectively). Cells were then washed once with PBS and subsequent experiments were carried out as described in the corresponding sections.

### 2.4. Scratch Assay

To measure the migratory capacity, cells were seeded with a density of 15,000 cells/cm^2^ in a 2-well silicone insert (Ibidi, Munich, Germany) with a defined cell-free gap (500 µm). For the experimental group, cells were exposed to hypoxic conditions (1% O_2_, 5% CO_2_, 94% N_2_, 37 °C) for 72 h. Images were taken immediately after removal of the insert (0 h), at 24 h, and at 72 h (C2C12-H cells or C2C12-*Mstn^−/−^*-H cells, respectively). In the control group (C2C12-C cells or C2C12-*Mstn^−/−^*-C cells, respectively), cells were cultured at normoxia, and images were taken at 0, 24, and 72 h as well. A bright field microscope (Zeiss Axiovert 100, Zeiss, Germany) with the following settings (Axiovision 4.8) was used: objective, 2.5×; exposure time, 614 ms; dimensions, 3900 × 3090 Px; scanned color. For evaluation of proliferation, the cell-free space was calculated, and the gap size was set in relation to the initial cell-free space.

### 2.5. Fluorescein Diacetate/Propidium Iodide (FDA/PI) Stain

For evaluation of cell viability, a live/dead stain was performed with FDA and PI. Cells were seeded with a density of 15,000 cells/cm^2^. After 24 h, HR was induced as described in Section 2.3. Subsequently, a staining solution of cell culture medium without FCS (5 mL) was mixed with FDA (8 µL; 5 mg/mL) and PI (50 µL; 2 mg/mL). After incubation for 5 min, cells were washed with PBS and analyzed with a fluorescent microscope (Olympus IX3-Series).

### 2.6. CellRox Green Assay

Cells were seeded in chamber slides with a density of 15,000 cells/cm^2^. After 24 h, HR was induced as described in Section 2.3. Then, a CellROX-Green Reagant Array was performed according to the manufacturer’s instructions (Molecular Probes™ CellROX Green Reagent, C10444, Eugene, OR, USA). Cells were then analyzed with a fluorescent microscope (Olympus IX3-Series).

### 2.7. Immunofluorescence

After HR, cells were washed with PBS for 15 min and subsequently fixed using 4% paraformaldehyde (PFA) for 30 min. After a blocking step (2% BSA at room temperature), cells were washed and incubated with a primary antibody overnight at 4 °C. Next, cells were washed, incubated with an appropriate secondary antibody for 1 h at room temperature, and afterwards counterstained with DAPI. After applying fluorescence-preserving mounting medium, images for immunofluorescence were taken with a fluorescence microscope (Olympus IX3-Series, Tokyo, Japan). By using the Adobe Magic Wand Tool (settings: tolerance, 60%; noncontiguous) immunocytochemical positive stained pixels were selected automatically and divided by countable nuclei. Afterwards, a mean value was calculated. A detailed list of used antibodies can be found in the supplemental material (Supplementary Table S1).

### 2.8. Statistics

After testing for homo- or heteroscedasticity (F-Test), p-values for pairwise comparisons were analyzed via a two-tailed unpaired t-test with (heteroscedasticity) or without (homoscedasticity) Welch’s correction. Multi-group comparisons were carried out using ANOVA followed by Tukey’s post-hoc test (homoscedasticity) or Brown–Forsythe and Welch ANOVA followed by Dunnett’s T3 post-hoc test (heteroscedasticity). All analyses were performed using GraphPad PRISM (version: 8.3.0, Graphpad Software, Inc., San Diego, CA, USA). Statistical significances were set at a *p*-value < 0.05. For figure preparation, Adobe Illustrator (version 24.1.1., Adobe Inc., San Jose, CA, USA) was used.

### 2.9. Study Approval

The authors certify that they comply with the ethical guidelines for authorship and publishing.

## 3. Results

### 3.1. Generation of a C2C12-Mstn^−/−^ Cell Line

**Figure 1:**
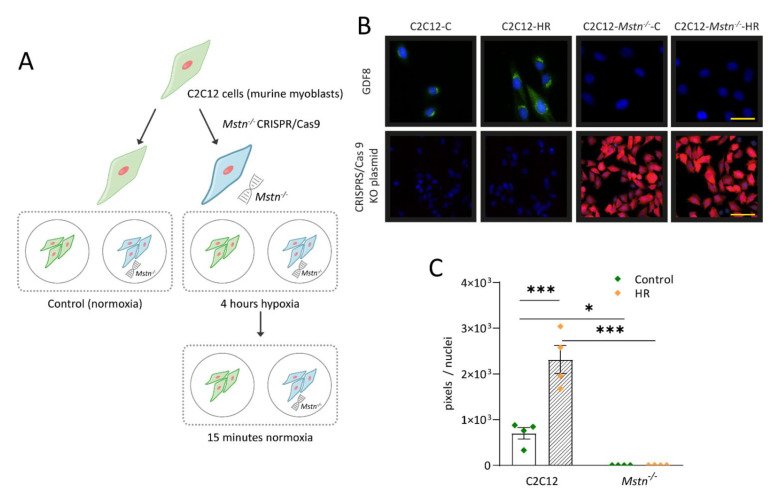
Schematic representation of the in vitro workflow and proof of successful gene editing via CRISPR/Cas9. (**A**) C2C12 cell genome was edited using CRISPR/Cas9 technology to induce *Mstn* knockout. C2C12 and C2C12-*Mstn^−/−^* cells were then exposed to 4 h of hypoxia, followed by 15 min of reoxygenation (HR). Control C2C12 cells were incubated at normoxic conditions for the same time. (**B**) The upper panel illustrates myostatin expression levels (*green*). The lower panel depicts a visualization of the CRISPR/Cas9 KO plasmid *(red)*. Cell nuclei were counterstained with DAPI. (**C**) Quantification of myostatin expression levels. C, control cells incubated under normoxia. HR, cells incubated under hypoxic conditions for 4 h followed by normoxia for 15 min. For each graph, four independent experiments per group were conducted. Scale bar = 25 µm (upper panel), 50 µm (lower panel). Results are shown as means ± SEM. *p*-value: * < 0.05, *** < 0.001; ANOVA followed by a multiple test comparison via Tukey’s post-hoc test (homoscedasticity) or Brown–Forsythe and Welch ANOVA followed by Dunnett’s T3 post-hoc test (heteroscedasticity).

In order to investigate mechanisms of IR signaling, we used CRISPR/Cas9 gene editing to induce a *Mstn* knockout in C2C12 cells. Cells were exposed to hypoxia for 4 h followed by reoxygenation for 15 min. Control cells were cultured at normoxia. Figure 1A illustrates the workflow used for HR experiments. Verification of successful gene editing was achieved using immunofluorescence myostatin staining (Figure 1B). C2C12-*Mstn^−/−^* cells displayed no measurable expression level of myostatin under normoxic and hypoxic conditions (Figure 1C). C2C12 cells that had not been gene edited showed myostatin activity in control cells and increased levels of myostatin upon HR (3-fold, *p* < 0.001). All in all, data were in line with the previously described interplay between IR/HR stress and myostatin and verified successful gene editing [21].

### 3.2. C2C12-Mstn^−/−^ Cells Are Less Susceptible to HR Injury In Vitro

**Figure 2:**
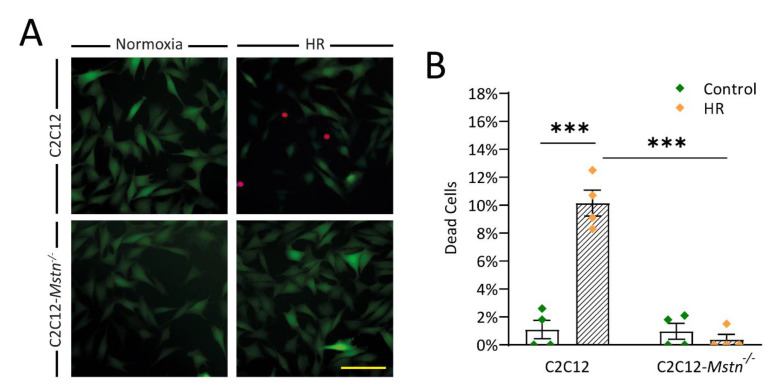
FDA/PI-Stain illustrates the better survival of C2C12-*Mstn^−/−^* cells when exposed to HR. (**A**) Representative images at 40× magnification displaying viable cells and dead cells under normoxic (left panels) and HR (right panels) conditions. (**B**) Graph depicting the percentual number of dead cells (Fluorescein Diacetate/Propidium Iodide stain) in C2C12 and C2C12-*Mstn^−/−^* cells under normoxic conditions and after HR. C, control cells incubated under normoxia. HR, cells incubated under hypoxic conditions for 4 h followed by normoxia for 15 min. For each graph, four independent experiments per group were conducted. Scale bar = 50 µm. Results are shown as means ± SEM. *p*-value: *** < 0.001; ANOVA followed by a multiple test comparison via Tukey’s post-hoc test (homoscedasticity) or Brown–Forsythe and Welch ANOVA followed by Dunnett’s T3 post-hoc test (heteroscedasticity).

After validating our in vitro model, we first sought to investigate overall cell survival in C2C12 and C2C12-*Mstn^−/−^* cells after being exposed to HR. This was achieved utilizing Fluorescein Diacetate/Propidium Iodide stain (Figure 2A). Propium Iodide can only permeate dead cells and can thus be used to assess cell viability. The number of dead cells rose from 1% to 10% (*p* < 0.001) regarding C2C12 cells (Figure 2B). Changes were also highly significant when comparing C2C12 cells and C2C12-*Mstn^−/−^* cells after HR. In C2C12-*Mstn^−/−^* cells, no increased cell mortality was observed.

### 3.3. Mstn Inhibition Mitigates HR-Induced Activation of the Intrisic Apoptotic Pathway

**Figure 3:**
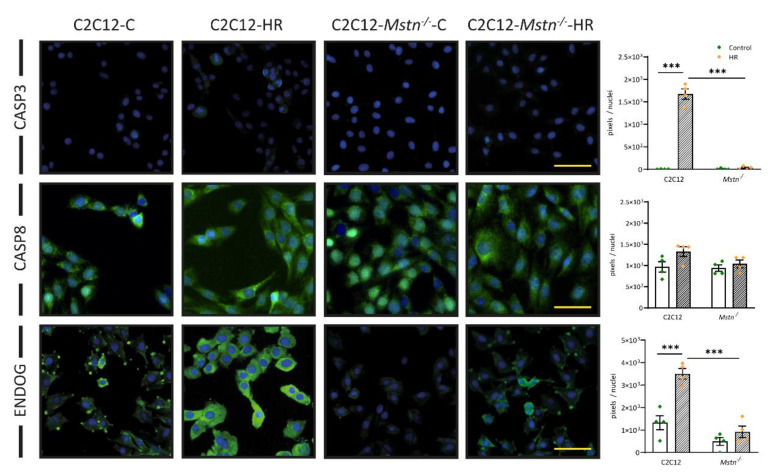
Expression levels of key proteins in intrinsic and extrinsic activation of apoptosis. Immunofluorescence analysis of intrinsic activation (caspase 3 and endonuclease G) and extrinsic activation (caspase 8), accompanied by representative images. C, control cells incubated under normoxia. HR, cells incubated under hypoxic conditions for 4 h followed by normoxia for 15 min. Cell nuclei were counterstained with DAPI. For each graph, four independent experiments per group were conducted. Scale bar = 50 µm. Results are shown as means ± SEM. CASP3, caspase 3; CASP8, caspase 8; ENDOG, endonuclease G. *p*-value: *** < 0.001; ANOVA followed by a multiple test comparison via Tukey’s post-hoc test (homoscedasticity) or Brown–Forsythe and Welch ANOVA followed by Dunnett’s T3 post-hoc test (heteroscedasticity).

Based on the FDA/PI stain results, we wanted to further investigate the intracellular processes in HR. Therefore, we determined the protein levels of caspase 3, caspase 8, and endonuclease-G (Figure 3). Caspase 3 is an executioner caspase involved in both the intrinsic and extrinsic pathway of apoptosis [8]. The extrinsic pathway is mainly activated via death ligands and subsequent caspase 8 activation [9]. The intrinsic pathway, on the other hand, is mainly initiated in response to mitochondrial damage [10]. We found caspase 3 levels to be highly upregulated after HR in C2C12 cells (451-fold, *p* < 0.001) but not in C2C12-*Mstn^−/−^* cells (3-fold, *p* > 0.05). Interestingly, caspase 8 levels did not exhibit a significant increase in C2C12 or C2C12-*Mstn^−/−^* cells after HR. Rather, immunofluorescence analysis of endonuclease G levels identified a 2.6-fold increase in C2C12-HR cells without any significant change in C2C12-*Mstn^−/−^*-HR cells when compared with control cells. Endonuclease G is a mitochondrial enzyme localized in the intermembrane space and recognized as a marker of apoptosis induced by mitochondria [22]. Therefore, the presented data suggest a reduced activation of the apoptosis surrogate marker caspase 3 in response to HR as a result of myostatin inhibition, potentially caused by mitigation of mitochondrial damage.

### 3.4. HR Induces Signaling in the Myostatin–pp38 axis That May Sidestep Regulation by pMEK3/6 in C2C12-Mstn^−/−^ Cells

**Figure 4:**
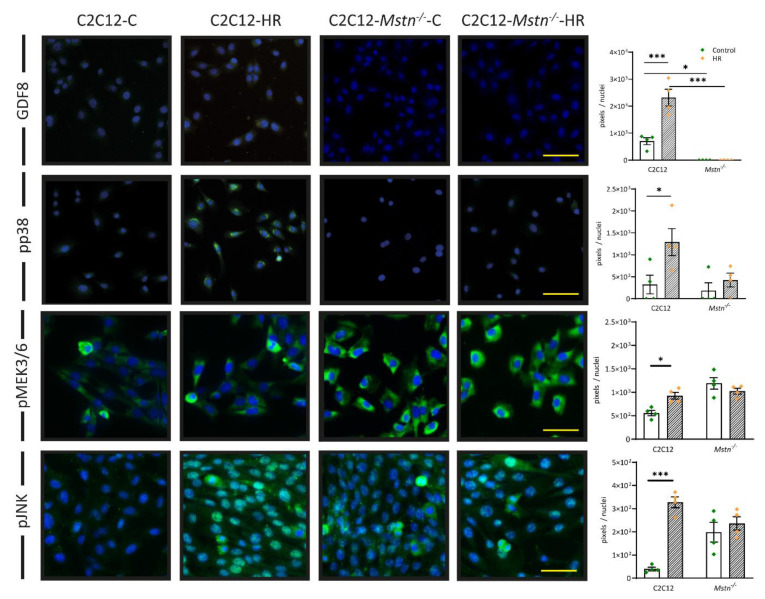
Intracellular signaling via the myostatin (GDF8)–pp38 axis, which sidesteps regulation via pMEK3/6 in C2C12-*Mstn^−/−^* cells. Immunofluorescence analysis of the myostatin–pp38 MAPK pathway, pMEK3/6, and pJNK. C, control cells incubated under normoxia. HR, cells incubated under hypoxic conditions for 4 h followed by normoxia for 15 min. For each graph, four independent experiments per group were conducted. Scale bar = 50 µm. Results are shown as means ± SEM. Cell nuclei were counterstained with DAPI. *p*-value: * < 0.05, *** < 0.001; ANOVA followed by a multiple test comparison via Tukey’s post-hoc test (homoscedasticity) or Brown–Forsythe and Welch ANOVA followed by Dunnett’s T3 post-hoc test (heteroscedasticity).

To further investigate the intracellular processes in response to HR, we analyzed protein levels of the myostatin–pp38 axis. In C2C12 cells, HR resulted in a 3-fold increase in myostatin without any signal nor enhancement in C2C12-*Mstn^−/−^* cells (Figure 4, first panel). Likewise, pp38-MAPK was upregulated in C2C12-HR cells compared with C2C12-C (4-fold, *p* < 0.05) but not in cells deficient in functioning myostatin (Figure 4, second panel). Strikingly, pMEK3/6 levels (Figure 4, third panel) increased in C2C12 cells as a result of HR (2-fold, *p* < 0.05) while pMEK3/6 levels were elevated in C2C12-*Mstn^−/−^* cells before HR but did not increase after HR. Phosphorylated MEK3 and MEK6 are kinases that phosphorylate p38 in response to stress stimuli as elicited by oxidative stress due to HR. Another mitogen-activated protein kinase besides p38 is c-Jun N-terminal-kinase (JNK). Together, p38 and JNK are responsible for the majority of intracellular signaling events activated upon cell stress and inflammatory cytokine activity [12,23]. Similar to p38 and pMEK3/6, levels of pJNK increased upon HR in C2C12-cells (8-fold, *p* < 0.001). In *Mstn^−/−^* cells, pJNK levels were elevated before HR when compared with C2C12 cells but did not increase upon HR. Taken together, the presented data suggest higher basal levels of intracellular signaling activity and cell stress, respectively, in C2C12-*Mstn^−/−^* cells, which may blunt their susceptibility to HR.

### 3.5. Inhibiting Myostatin Signaling Lowers Early Lipid Peroxidation and Nitrosative Stress Albeit ROS Generation in General Is Equal in C2C12 and C2C12-Mstn^−/−^ Cells after HR

**Figure 5:**
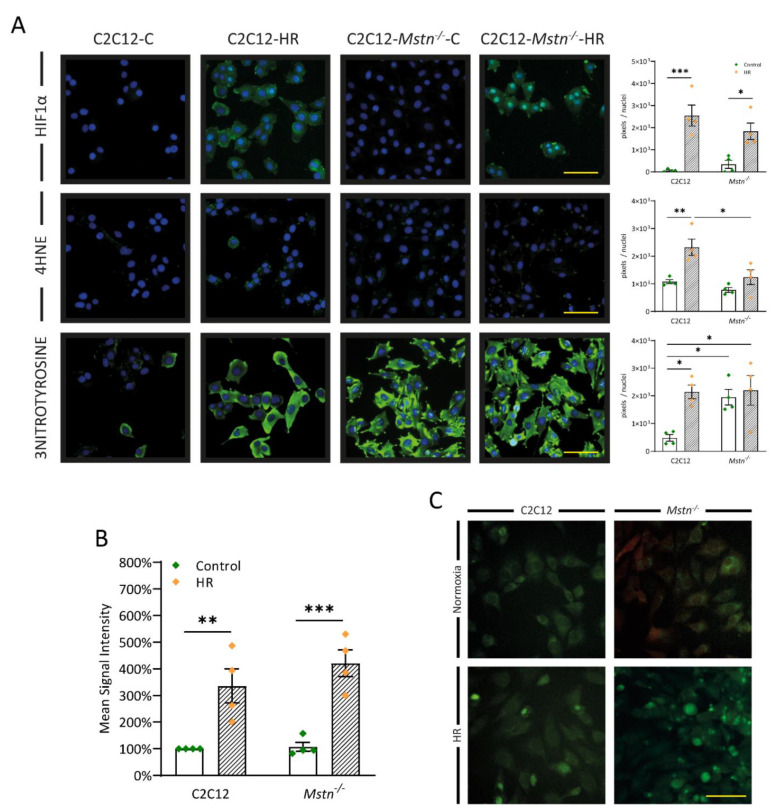
Rise of 3-Nitrotyrosine and 4-Hydroxynonenal (4HNE) levels due to HR in C2C12 cells accompanied by equal levels of ROS before and after HR in C2C12 and C2C12-*Mstn^−/−^* cells. (**A**) Immunofluorescence analysis of markers of hypoxia (HIF-1α), early lipid peroxidation (4-HNE), and nitrosative stress (3-Nitrotyrosine). Cell nuclei were counterstained with DAPI. (**B**) ROS formation in C2C12 and C2C12-*Mstn^−/−^* cells visualized and quantified using CellROX green reagent (Thermo Fisher Scientific, Massachusetts, USA). (**C**) Representative images of ROS visualization under normoxic (upper panel) and HR (lower panel) conditions. C, control cells incubated under normoxia. HR, cells incubated under hypoxic conditions for 4 h followed by normoxia for 15 min. For each graph, four independent experiments per group were conducted. Scale bar = 50 µm. Results are shown as means ± SEM. *p*-value: * < 0.05, ** < 0.01, *** < 0.001; ANOVA followed by a multiple test comparison via Tukey’s post-hoc test (homoscedasticity) or Brown–Forsythe and Welch ANOVA followed by Dunnett’s T3 post-hoc test (heteroscedasticity).

Regarding the pathophysiology and cell signaling of IR, an important role is attributed to reactive oxygen species (ROS) [24,25]. Measuring ROS activity (Figure 5B,C) using a CellROX reagent assay did not show any differences between C2C12 and C2C12-*Mstn^−/−^* cells. Both cell lines exhibited a strong enhancement of the mean signal intensity upon HR leading to the conclusion that myostatin deficiency is not capable of inhibiting ROS generation. Additionally, C2C12 and C2C12-*Mstn-^−/−^* cells sensed hypoxic conditions, which was reflected by significantly enhanced HIF-1α levels after HR in both groups (Figure 5A, first panel). Besides ROS, downstream cascades of IR injury are also triggered by generation of reactive nitrogen species (RNS) [26]. RNS, e.g., peroxynitrite, derive from •NO and O_2_^•−^ via NOS2 and NADPH oxidase [27]. Because RNS are difficult to measure, 3-nitrotyrosine (3NT) acts as surrogate marker for nitrosative stress [28]. Due to HR, 3NT increased in C2C12 cells (4-fold, *p* < 0.05, Figure 5A, third panel). In C2C12-*Mstn^−/−^* cells, 3NT did not display significant changes upon HR. However, the baseline activity before and after HR approximately equaled 3NT levels in C2C12 cells after HR. Despite these findings, C2C12-*Mstn^−/−^* cells demonstrated no statistically significant increase in 4-Hydroxynoneal (4HNE) due to HR (*p* = 0.423) while C2C12 cells displayed a 2.3-fold increase (*p* < 0.01, Figure 5A, second panel), suggesting that myostatin inhibition mitigates early lipid peroxidation in vitro.

### 3.6. C2C12-Mstn^−/−^ Cells Exhibit Increased Cell Migration under Hypoxic Conditions While C2C12 Cells Demonstrate Diminished DNA Replication

**Figure 6:**
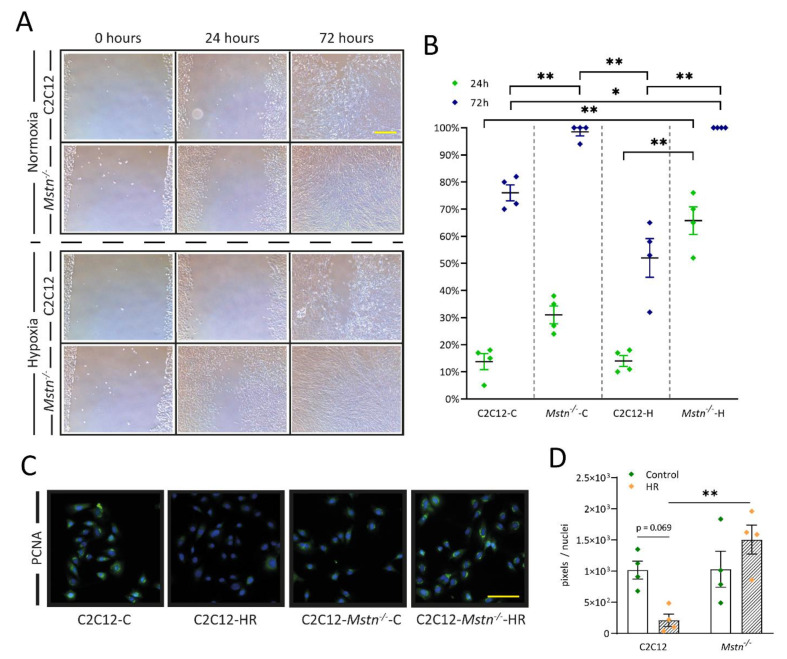
Elevated proliferation of C2C12-*Mstn^−/−^* cells under hypoxic conditions and measurement of DNA replication. (**A**) Images of the scratch array measuring the temporal progression of cell migration and proliferation under normoxic and hypoxic conditions. (**B**) Graph depicting percentual coverage (x-axis) of the initial defined gap after 24 h (green symbols) and 72 h (blue symbols) for C2C12 and C2C12-*Mstn^−/−^* cells under normoxic (**C**) and hypoxic (H) conditions. (**C**) Representative images at 40× magnification and (**D**) immunofluorescence analysis of proliferative activity (PCNA). Cell nuclei were counterstained with DAPI. C, control cells incubated under normoxia. H, cells incubated under hypoxic conditions for 72 h. HR, cells incubated under hypoxic conditions for 4 h followed by normoxia for 15 min. For each graph, four independent experiments per group were conducted. Scale bar = 50 µm. Results are shown as means ± SEM. *p*-value: * < 0.05, ** < 0.01; ANOVA followed by a multiple test comparison via Tukey’s post-hoc test (homoscedasticity) or Brown–Forsythe and Welch ANOVA followed by Dunnett’s T3 post-hoc test (heteroscedasticity).

Given the increased survival capacity under HR conditions, we also sought to investigate cell proliferation in temporal progression. Cell proliferation and migration were measured using a scratch assay (Figure 6A). After seeding and removing the insert, cell migration was analyzed after 24 and 72 h (Figure 6B). Except for a brief analysis under a microscope at given time points, cells were exclusively cultured at hypoxia (1% O_2_). Strikingly, *Mstn^−/−^* cells migrated substantially faster under hypoxic conditions (*Mstn^−/−^*-H cells) compared with C2C12-H cells (*p* < 0.01) and C2C12 control cells (C2C12-C, *p* < 0.01) at 24 h. C2C12 cells with functioning myostatin were essentially more prone to the hypoxic environment. At 72 h, C2C12-*Mstn^−/−^*-C and C2C12-*Mstn^−/−^*-H cells demonstrated full confluency, which is an impressive finding, considering that C2C12 cells only displayed 76% confluency under normoxic conditions. Under hypoxic conditions, this number was lowered to 52%. Increased proliferative capacity in C2C12-*Mstn^−/−^* cells was also verified using PCNA stain. Due to hypoxia, levels in C2C12 cells were reduced by 79% (*p* = 0.069) while levels in C2C12-*Mstn^−/−^* cells increased slightly but nonsignificantly.

## 4. Discussion

In this work, we could validate previously described protective effects of myostatin deficiency in HR in skeletal muscle cells in vitro and investigated their potential mechanisms. Using a gene-edited C2C12 cell line, FDA/PI-stain demonstrated a lower rate of cells that died in response to HR. In subsequent experiments, we detected significantly less apoptotic activity upon HR in C2C12-*Mstn^−/−^* cells. In this regard, we found apoptosis in response to HR to be mainly transduced through the intrinsic branch of the cascade, which is in line with previous studies [29]. Caspase 8, an effector caspase of the extrinsic pathway, that is classically activated via death receptors, did not show significantly increased levels after HR in either group. This has also been described earlier [29], although some authors have stated mitigation of ischemia reperfusion due to, inter alia, caspase 8 inhibition [30]. One possible explanation is the rather short time frame of reoxygenation in our study. The role of caspase 8 and the extrinsic apoptosis pathway in IR injury is usually attributed to the later reperfusion stages [31]. Therefore, increased caspase 3 activity is very likely explained by augmented stimulation of the intrinsic pathway, as suggested by other authors. This is supported by increased endonuclease G levels in C2C12 after HR. Endonuclease G is a protein located in the intermembrane mitochondrial space [22]. While mitochondria are the origin of intrinsic apoptosis, recent studies have shown that endonuclease G is also capable of DNA degradation without caspase activation [32]. One could therefore hypothesize that myostatin deficiency not only protects muscle cells from the intrinsic apoptotic activity seen in early IR/HR injury but also halts DNA degradation independent of caspase activity. The scratch assay and PCNA stain results further support this claim. Additionally, it should be emphasized that C2C12-*Mstn^−/−^* cells grew faster in response to HR than under normoxic conditions. Investigation of intracellular signaling that conveys protective effects of myostatin deficiency also led to new insights. It is well known that myostatin mainly signals through SMAD2/3 and p38. The latter is one of the main reactive proteins in response to cellular stress (e.g., hypoxia, ROS formation) [12,33] and constitutes the overlap between myostatin signaling and IR/HR. This has also been investigated in previous studies [14,34]. In the context of cellular stress, p38 MAPK itself is mainly activated via phosphorylation by MEK3 and MEK6 [13,35]. Therefore, absence of heightened p38 phosphorylation suggests MEK3/6 as a potential mediator of protective effects of myostatin deficiency. Another novelty of this study is the lack of an increase in JNK signaling due to HR. Previous work has mainly focused on the p38 pathway when exploring protective effects of myostatin deficiency. Here, we identified JNK to play a potential role as well. Lastly, we investigated the formation of ROS and RNS. Although nitrosative stress and lipid peroxidation were reduced, overall ROS formation was equal. Pro-oxidant properties of myostatin inhibition as well as concurrently decreased lipid peroxidation have been elucidated earlier [18,36]. When evaluating decreased lipid peroxidation, on the other hand, one should consider the altered skeletal muscle cell lipid composition of myostatin-deficient cells. As previously shown, this can potentially lead to generally decreased lipid oxidation [37]. Nevertheless, ROS levels as measured with CellRox were equal in both groups after HR, suggesting that the protective capacity of myostatin deficiency in the pathophysiological cascade of HR/IR is rather located between ROS generation and mitochondrial and subsequent DNA damage. Another possible conclusion that can be drawn from this study is that myostatin deficiency may lead to overall enhanced cell proliferation and cell turnover. Besides the unaffectedness of cell proliferation illustrated by PCNA stain and increased cell migration in the scratch assay, C2C12-*Mstn^−/−^* cells have exhibited higher basal protein levels with non-reactiveness to HR in the case of pMEK3/6, pJNK, 3NT, and 4HNE. One could speculate that stress signaling is increased in myostatin deficiency states which, to an extent, conditions muscle cells to be less susceptive to HR. Indeed, remote ischemic preconditioning has been thoroughly investigated [1,27] and there are clinical trials that explore their benefit in, for instance, human coronary artery bypass surgery [38]. When investigating HR/IR injury, there are some inherent limitations. First, mechanisms of cell death in the setting of HR/IR injury are versatile. In this research, we mainly focused on the role of apoptosis as the most thoroughly investigated form of programmed cell death. Certainly, mechanisms such as necrosis and necroptosis [39] have gained attention over the last few years and may be important aspects with a lot of potential for further research. Additionally, experiments regarding oxidative stress did not show entirely conclusive results. Therefore, generation of nitrosative stress and ROS generation in the setting of myostatin deficiency require additional investigation. Finally, the significance of the results could have been further improved by including non-targeting RNA or empty vectors in the control group to rule out a potential bias caused by CRISPR/Cas9 gene editing. Collectively, the data in these experiments validated protective effects of myostatin deficiency in vitro and provided insights into intracellular signaling in the setting of HR. The absence of functioning myostatin did not inhibit ROS formation but did lower apoptotic activity, particularly the intrinsic branch that originates from mitochondria. Furthermore, inhibition of proliferation due to HR injury was stronger in cells not deficient in myostatin. This study may further stimulate research that focuses on the role of p38, MEK3/6, and JNK when investigating intracellular signaling in HR/IR.

## Data Availability

The data presented in this study are available on reasonable request from the corresponding author.

## Supplementary Materials

The following are available online at https://www.mdpi.com/article/10.3390/cells10071680/s1: Table S1: List of Antibodies.
