# Supplementary material for: Myostatin Deficiency Protects C2C12 Cells from Oxidative Stress by Inhibiting Intrinsic Activation of Apoptosis
Source: Cells. 2021 Jul 3;10(7):1680. doi: 10.3390/cells10071680 (PMC8305813; doi:10.3390/cells10071680)
Supplement: Supplementary file 1 [file cells-10-01680-s001.zip › cells-1261067-supplementary.pdf]

Antibody	Company	Type	Concentration	Catalogue
GDF-8/11 (H-9)	Santa Cruz	Rabbit polyclonal	1:50	sc-393335
p-p38 MAPK (T180/Y182) (D3F9)	Cell Signaling	Rabbit monoclonal	1:500	4511S
HIF-1α	Abcam	Rabbit monoclonal	1:50	ab179483
Caspase 3 (H-277)	Santa Cruz	Rabbit polyclonal	1:50	sc-7148
4- Hydroxynonenal	Abcam	Rabbit polyclonal	1:200	ab46545
3- Nitrotyrosine (39B6)	Santa Cruz	Mouse monoclonal	1:200	sc-32757
Endonuclease G (B-2)	Santa Cruz	Mouse monoclonal	1:200	sc-365359
PCNA (PC10)	Santa Cruz	Mouse monoclonal	1:100	sc-56
p-JNK (G-7)	Santa Cruz	Mouse monoclonal	1:100	sc-6254
p-MEK-3/6 (B-9)	Santa Cruz	Mouse monoclonal	1:100	sc-8407
Caspase 8 (1.1.40)	Santa Cruz	Mouse monoclonal	1:100	sc-81656
Alexa Fluor 594 anti-rabbit	Alexa Fluor	Goat anti rabbit	1:1000	A11012
Alexa Fluor 488 anti-rabbit	Alexa Fluor	Goat anti rabbit	1:1000	A11008
m- IgGk BP-CFL 488	Santa Cruz Biotechnologie	IgGκ binding protein	1:200	sc-516176
